# A Psychological Profile of Elite Polish Short Track Athletes: An Analysis of Temperamental Traits and Impulsiveness

**DOI:** 10.3390/ijerph19063446

**Published:** 2022-03-15

**Authors:** Katarzyna Gabrys, Antoni Wontorczyk

**Affiliations:** 1 Doctoral School in Social Sciences, Faculty of Management and Social Communication, Jagiellonian University, 30-348 Krakow, Poland; 2 Institute of Applied Psychology, Faculty of Management and Social Communication, Jagiellonian University, 30-348 Krakow, Poland; antoni.wontorczyk@uj.edu.pl

**Keywords:** skating, temperament, impulsivity, individual differences, professional athletes

## Abstract

The aim of this study was to determine the temperament and impulsiveness profile of short track athletes. Professional athletes (juniors and seniors), under training in the Polish National Team (N_female_ = 21, N_male_ = 19, M_age_ = 20), completed The Temperament and Character Inventory- Revised (TCI-R (56)) and a shortened version of the Urgency, Premeditation, Perseverance, Sensation-Seeking, Positive Urgency, Impulsive Behavior Scale (S-UPPS-P). The results proved that skaters obtain higher scores than the general population on the temperamental scales i.e., persistence, harm avoidance and novelty seeking and impulsivity scales i.e., sensation seeking and positive urgency. After the cluster analysis, two homogeneous profiles of short track athletes were determined. The first profile includes athletes with high scores on the reward dependence, persistence, self-directedness, cooperativeness, temperamental and sensation-seeking impulsiveness scales coupled with low scores on the temperamental scale, harm avoidance and impulsiveness scales: positive urgency, negative urgency and the lack of perseverance. The second profile is the reverse of the first profile for the short track athletes.

## 1. Introduction

Short track is one of the most recent winter sports. It has been gradually evolving from a niche sport to become increasingly more popular. The availability of venues on which it can be practiced plays a significant role in the sport gaining popularity. Short track competitions and trainings are held on a track, which is set on an ice sheet, which means that they can take place on any ice rink adapted for ice hockey [1]. It is also a very attractive sport for the audience, as it is unpredictable and risky. Four to six competitors start from a single line, and remain in direct contact during the entire race. Skaters can reach speeds of up to 50 km/h. This makes short track a risky discipline with a high injury rate [2].

Previous research on short track athletes has focused on the athletes’ physical preparation [3,4] and their physical skills [5]. Besides speed preparation, speed and strength endurance [6,7,8], perfection of skating tactic and technique [9] are also important determinants of athletic performance. However, researchers have begun to concentrate on psychological skills, i.e., coping, anxiety [10], anger [10,11], mental toughness [12] and personality traits [13]. Considering, how important psychological skills are in an athlete’s preparation [14,15], it seems that physical skills are insufficient to become a highest level athlete.

Researchers are looking more at athletes of different sports, through the context of their psychological and dispositional variables [16]. To date, several studies have investigated psychological profiling based on emotions [17], personality traits [18,19] and temperament [20,21,22] as well as temperamental traits in athletes of different sports [23,24,25,26,27] or correlations between psychological profile and competitive anxiety, moods and self-efficacy [28]. The integration of qualitative and quantitative characteristics in profiling is also being investigated [29]. However, these studies have not considered temperamental traits in short track athletes, which makes them a population worth testing. Temperamental traits determine the choice in forms of activity of professional and sport character, which have a varied stimulus value [30]. This is especially relevant for forms of activity that involve high situational tension on both time and energy [31], such as short track. 

According to the psychobiological theory by Cloninger et al. [32], temperament is the biological basis for the development of personality traits and can be understood as a genetically determined set of skills and emotional reactions to the environmental stimuli. Temperament can be described on four dimensions: novelty seeking (NS), harm avoidance (HA), reward dependence (RD) and persistence (P). The four-dimensional model has been extended to a seven-dimensional model, including three dimensions of character, in order to better represent individual differences [32,33]. The character dimensions are self-directedness (SD), cooperativeness (C) and self-transcendence (ST) [32]. Taking into account the importance of temperament as a factor that, in a task situation, determines human behavior, and therefore determines the form of activity [30], it can be assumed that it will determine the choice in the practiced sport discipline. It is worth noting that individuals with similar temperament may manifest different behaviors depending on how their character traits have developed [32]. Thus, how we guide an individual’s personality development is important. A variable such as temperament, therefore, seems particularly significant in the context of finding young, talented athletes [34] and preparing an even more personalized training.

As mentioned, short track is a highly injury-prone discipline, with up to 64.2% of athletes suffering at least one injury during the competitive season [35]; hence, it is also a risky sport. Until now, investigations of impulsiveness, in the field of sport psychology, have indicated a correlation between impulsiveness and athletes’ disposition to risky behavior [36]. Keeping this in mind along with the characteristics of the discipline, impulsiveness seems to be an important dispositional variable of a short track athlete. Impulsiveness can be understood as the predisposition to react rapidly, abruptly, and in an unplanned manner to internal and external stimuli to take action [37]. A study by Whiteside and Lynam [38] indicated that there are four personality pathways that lead to impulsiveness: urgency, lack of premeditation, lack of perseverance and sensation seeking. The urgency, as evidenced by the research of Cyders et al. [39], includes two aspects: positive and negative urgency. Negative urgency is the tendency to engage in impulsive behavior under the influence of negative emotions, whereas positive urgency is the tendency to engage in impulsive behavior under the influence of positive emotions.

Considering the specifics of the discipline, we believe that short track athletes would be characterized, not only by specific physical, but also temperamental and impulsivity traits. The aim of this study was to determine the temperament profile of short track athletes. The hypothesis which will be tested, is that short track athletes are characterized by a low level of harm avoidance (HA), especially because the discipline they practice is highly unpredictable and injury prone [2]. We also hypothesize that the short track athletes would be characterized by a low level of novelty seeking (NS), due to the fact that performing a sport professionally requires patience, and a tolerance for delayed gratification [32,33]. Furthermore, the level of persistence (P) would be high due to the tendency of professional athletes to be perseverant, persistent and stable despite frustration [40]. In the impulsiveness context, we hypothesize that short track athletes would have increased scores on the sensation-seeking and positive urgency scales. Given the results of previous studies, which discuss the issue of sex-based differences [41,42,43,44], we can hypothesize that in the study group, female athletes will score higher than male athletes on the HA, RD and C scales. We further hypothesize sex-based differences in the athletes’ temperament profiles. In a correlation context, we assume a negative correlation between persistence and lack of perseverance, because these variables exclude each other. We suppose that the cluster analysis would enable the determination of one temperament profile of the short track athlete.

## 2. Materials and Methods

### 2.1. Design

The data obtained in the study were analyzed by descriptive and inferential statistical analysis. The distribution of quantitative variables in terms of normality was analyzed by using skewness, kurtosis and Kolmogorov–Smirnov tests. It was assumed that if skewness was between −1 and 1, kurtosis was between −2 and 2, and also the result of the Kolmogorov–Smirnov test were not statistically significant, the analyzed quantitative variables would then have a distribution that is close to normal [45]. Additionally, if a quantitative variable did not have a normal distribution, in order to adjust it to normality, it was adjusted using ln(xx) and x2. The obtained results allow the application of parametric tests in the statistical analysis of the variables. Internal reliability of particular scales was measured by Cronbach’s alpha. Its value ranged from 0.63 for the lack of premeditation (LPr) scale to 0.91 for the harm avoidance (HA) scale. Student’s *t*-test and two-dimensional Pearson’s test were used to analyze the variables. A k-means cluster analysis was used to determine dispositional profiles. The IBM SPSS Statistics 27, PS IMGO PRO 7.0 software package was used for statistical processing of the data.

### 2.2. Subjects

The sample consisted of 40 professional athletes (juniors and seniors), under training in the national team, i.e., the best athletes in Poland. The age of the athletes ranged between 18 and 33. The average age of the athlete was 20 years old. The research included female (*n* = 21) and male (*n* = 19) athletes. The research was carried out during the national team training camps.

The present study was approved by the Jagiellonian University Institute of Applied Psychology Research Ethics Committee (app. no. 107/2021). The study was conducted according to the guidelines of the Declaration of Helsinki [46,47,48]. The objectives of the study and its requirements were explained to the subjects, and all participants provided consent.

### 2.3. Assessment Tools and Data Collection

The following tests were used in the study: The Temperament and Character Inventory-Revised (TCI-R (56)) [49] (Polish adaptation Wontorczyk [50]) and a shortened version of the Urgency, Premeditation, Perseverance, Sensation-Seeking, Positive Urgency, Impulsive Behavior Scale S-UPPS-P [51] (Polish adaptation Poprawa [52]). The results of the study were subjected to statistical analysis.

The TCI-R (56) [49,50] is currently the most used questionnaire to investigate Cloninger’s personality model. The questionnaire has 56 items, eight for each of the seven dimensions it measures (four of temperament and three of character). The four dimensions of temperament are novelty seeking (NS), harm avoidance (HA), reward dependence (RD) and persistence (P). The three dimensions of character are self-directedness (SD), cooperativeness (C) and self-transcendence (ST). The subjects respond on a 5-point Likert scale ranging from 1 (definitively false) to 5 (definitively true). Scores for each dimension range from 1 to 40.

The S-UPPS-P [51,52] is used to investigate the personality pathways that lead to impulsiveness. The questionnaire has 20 items, four for each of the five dimensions it measures. The five dimensions of impulsiveness are positive urgency (PU), negative urgency (NU), lack of premeditation (LPr), lack of perseverance (LP) and sensation seeking (SS). The subjects respond on a 4-point scale ranging from 1 (definitively true) to 4 (definitively false). The scores for each dimension range from 1 to 16.

## 3. Results

As was mentioned in the introduction, to describe the dispositional profiles of short track athletes, we used Clonninger’s model [32], which allows the diagnosis of temperamental and character traits. Table 1 shows that the athletes scored the highest only on the persistence (P) scale (X = 27.43; SD = 5.51). The lowest score was obtained on the novelty-seeking scale (X = 2.95; SD = 3.98). The athletes obtained average scores on the remaining two scales that measure temperamental traits (harm avoidance and reward dependence). As far as character traits are concerned, the highest score was obtained on cooperativeness (C) (X = 27.3; SD = 5.66), and the lowest on self-transcendence (ST) (X = 24.66; SD = 4.87). After considering the sex variable, the results for temperamental traits differed only in the case of harm avoidance (HA) (Z = 2.54; *p* = 0.01). Females scored higher than males on HA (males: X = 26.28, SD = 5.15; females: X = 21.57, SD = 4.91). Regarding character traits, statistically significant differences were obtained only for cooperativeness (C) (Z = 2.07, *p* = 0.03). C female athletes also scored higher than male athletes (females: X = 29.00, SD = 5.78; males: X = 25.42, SD = 5.01). For impulsiveness, the athletes obtained the highest score on the sensation-seeking (SS) scale (X = 11.87, SD = 2.48) and slightly lower but also high on negative urgency (NU) scale (X = 10.9, SD = 2.21). The lowest scores were obtained on the lack of perseverance (LP) scale (X = 7.62, SD = 2.31). On the remaining scales, the results were average, (Table 1). The athletes’ sex was not a differentiating factor in the athletes in terms of impulsiveness either. None of the five scale scores was statistically significant.

A further analysis showed relationships between the temperament and character scales as well as impulsiveness scales in the studied group. The results, presented in Table 2, show such co-variations. A strong negative correlation was found between persistence (P) and the lack of perseverance (LP) (r = −0.48; *p* = 0.001), and a strong positive correlation- between persistence (P) and sensation seeking (SS) (r = 0.47, *p* = 0.001). A strong correlation was also found between novelty seeking (NS) and then lack of premeditation (LPr) (r = 0.46, *p* = 0.001). On the other hand, weak but statistically significant correlations were identified between persistence (P) and negative urgency (NU) (r = −0.33, *p* = 0.03) as well as between self-transcendence (ST) and positive urgency (PU) (r = 0.33, *p* = 0.02).

**Figure 1:**
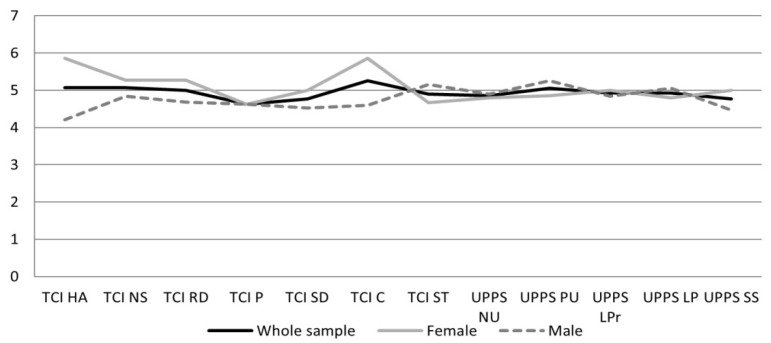
Short track athletes temperamental, character and impulsiveness profile, presented on the sten scale. Note: TCI-Temperament and Character Inventory; UPPS-Urgency, Premeditation (lack of), Perseverance (lack of), Sensation-Seeking, Positive Urgency, Impulsive Behavior Scale; HA-harm avoidance; NS-novelty seeking; RD-reward dependence; P-persistence; C-cooperativeness; SD-self-directedness; ST-self-transcendence; NU-negative urgency; PU-positive urgency; LPr-lack of premeditation; LP-lack of perseverance; SS-sensation seeking.

In the next step, it was decided to conduct a profile analysis on both TCI-R(56) and UPPS scale scores. For this purpose, the raw scores were transformed into STEN. Results between the first and fourth sten should be interpreted as low, results between the fourth and fifth sten, as average, and results above the fifth sten as high. The data are presented on Figure 1.

The analysis of the disposition profiles of short track athletes according to the disposition traits (temperament and character traits), shows that only cooperativeness, harm avoidance, and novelty seeking are in the high range. The profile obtained in the remaining scales, is within the range of the average result. As for the impulsiveness profile, the SS score is slightly above average. On other scales of impulsiveness, the short track athletes are characterized as average. The profiles, especially in relation to dispositional traits (temperament and character), are clearly different. Female athletes were characterized by high scores on the following scales: cooperativeness and harm avoidance, and slightly above average on: novelty seeking and reward dependence. Female athletes obtained average scores on the remaining scales. In the male profile, high scores were noted only on self-transcendence, and sensation-seeking scales, and slightly above average on the lack of perseverance. In the other dispositional traits of the male profile, the obtained results are average.

**Figure 2:**
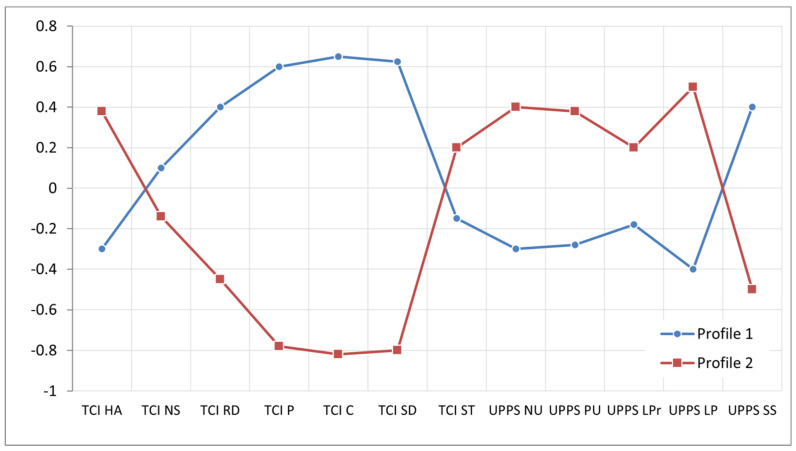
The temperament, character and impulsiveness profiles of short track athletes.

Multidimensional exploratory techniques and k-means cluster analysis were used to test the possibility of distinguishing a homogeneous group in terms of criterion variables (temperament, character and different forms of impulsivity). The results are shown in Figure 2 and Table 3.

The results of the cluster analysis with regard to the criterion traits (temperament, character and impulsivity traits) show that, in the group of the short track athletes, there are two homogeneous profiles (N_Profile1_ = 22; N_Profile2_ = 18). The profiles only do not differ for three scales: novelty seeking (F = 0.519, *p* = 0.47), self-transcendence (F = 0.994, *p* = 0.32) and the lack of premeditation (F = 1.305, *p* = 0.26). For the other scales, the results of the profiles are statistically, significantly different (Table 3). The first profile includes athletes with high scores on the temperamental scales: reward dependence, persistence, self-directedness, and cooperativeness and sensation seeking on the impulsiveness scale. Such athletes score low on the harm avoidance, positive urgency, negative urgency and the lack of perseverance. The second profile, in contrast, differs significantly from the first profile. It includes athletes with high scores on harm avoidance and positive urgency, negative urgency and the lack of perseverance. On the other hand, they have low scores on reward dependence, persistence, self-directedness, cooperativeness and sensation seeking.

## 4. Discussion

The aim of the study is to design a psychological profile of elite, Polish, short track athletes, based on their temperamental traits and impulsiveness. This study found that the short track athletes obtained the highest scores on the temperamental persistence scale (P). This means that they are persistent and stable individuals who pursue their goals despite frustration and fatigue [32]. This is consistent with our hypotheses and also with previous research on athletes in diverse disciplines [53]. According to H. Han et al. [53], winning athletes score higher on the P scale than losing athletes. In this study, the participants were athletes from the national team, in other words, athletes thought to be the best in the country. Short track athletes’ lowest scores were found on the novelty-seeking (NS) scale. This confirms the hypothesis, that professional sport requires patience and tolerance of delayed gratification, thus a low level of athletes’ NS. This is also consistent with a high score on the P scale. Individuals with a high level of NS quickly lose interest when their needs are not met [32]; this also precludes them from being goal-oriented people, which is characterized by a high level of P. However, this outcome is contrary to that reported by Parmigiani et al. [54], who found that winning athletes show higher levels of NS than losing athletes. The outcome might be contrary to the results of Parmigiani et al. [54] due to the different characteristics of the studied disciplines (combat sports, and skating). According to Sohrabi at al. [55], athletes who participate in contact sports are more aggressive and more likely to be engaged in risky behaviors than athletes in non-contact sports. Individuals with higher levels of aggression also have lower levels of patience and tolerance for delayed gratification [56]. In short track, contact with an opponent can lead to disqualification, so it can be considered as a non-contact sport, which may also explain the contradictory results. On scales related to character, short track athletes’ scores were highest on the cooperativeness (C) scale. This means, that they are empathetic and socially tolerant [32]. This result may be explained by the specificity of the discipline, as the athletes compete both individually and in relay races, which requires cooperation between athletes [57,58,59]. This finding may also be related to the discipline’s rules that require the athlete to pay attention to the other athletes to avoid getting in their way, for which one can be disqualified [60]. In addition, athletes adapt their pace to that of their opponents during the race [61]. The study shows the difference between female and male athletes in the HA and C levels, which were, in both cases, higher in females. This is consistent with our hypothesis and the results of other studies [41,42,43,44]. Only the level of the RD was not as we assumed, which means that there was no difference between females and males. In terms of impulsiveness, the hypothesis that short track athletes would score high on the sensation-seeking (SS) scale was confirmed. This result is not consistent with the athletes’ low scores on the NS temperamental scale. The model of temperament devised by Cloninger et al. [32] includes impulsiveness as an aspect of the NS scale. Therefore, the athletes’ simultaneous low scores on the NS and high scores on the SS scales are inconsistent. The athletes scored lowest on the lack of perseverance (LP) scale. This is logical, and consistent with simultaneously obtained high scores on the P scale of temperament. This study noted a strong negative correlation between these two variables, which is consistent with our hypothesis.

The results of the profile analysis show, that the elite short track athlete will have scores above average on the scales: C, HA, and NS. These results reflect those reported by Kang et al. [62], who also found that professional baseball players scored higher levels of C than non-players. Baseball is a team sport; however, short track also requires cooperation between the athletes on one team. Short track also requires the athletes to focus their attention on the opponents’ actions at all times. High levels of HA are characteristic of individuals with high levels of shyness and anxiety [32]. The high level of HA in short track athletes is consistent with other studies of athletes in different sports [53]. These results, reported by H. Han et. al. [53], show that HA scores are higher in athletes than non-athletes, and are particularly high in swimmers. Swimming is a close discipline to short track in terms of its competitive nature, as it is also race-based and includes team challenges. Individuals who have high NS scores quickly engage in new situations and make quick decisions [32]. H. Han et al. [53] showed that athletes in combat sports, and power/combat sports, scored higher on the NS scale than athletes in endurance, individual and team sports. In fact, the NS scale is close to the sensation-seeking (impulsiveness) scale. The research carried out by O’Sullivan et al. [63] indicates high levels of sensation-seeking behavior in body contact sports participants. During short track competition, athletes are in close contact with their opponents, which demonstrates the consistency of our results with previous studies of athletes [53,63]. The other scale of impulsiveness that is above average in elite short track athletes is positive urgency (PU). This means, that athletes will have a tendency to act rashly under extremely positive emotions [39].

Two homogeneous profiles of short track athletes were determined in this study, which did not correspond with our assumption. The first profile includes athletes with high scores on the reward dependence (RD), persistence (P), self-directedness (S), cooperativeness (C), temperamental and SS impulsiveness scales. Some athletes scored low on the temperamental scale, HA and impulsiveness scales: positive urgency (PU), negative urgency (NU) and lack of perseverance (LP). This means that they are individuals who will maintain previously reinforced behaviors, are hard working, and who persist in pursuing their goals despite fatigue [32]. Low HA scores may indicate that they will return to athletic activity quickly after injury, which seems significant in an injury-prone sport [2]. The profile is complemented well by a constellation of dispositional traits that Cloninger referred to as ‘organized character profiles’ [64,65,66]. It could be assumed that athletes with this profile are individualists, who can be high functioning, and who can work effectively with others if achieving an important goal (e.g., sport success) requires cooperation [67]. This profile is well matched to short track athletes. The second profile is the reverse of the first profile. It includes athletes with high scores on the HA temperamental scale, as well as on the PU, NU and LP impulsiveness scales. They have low scores on the RD, P, SD, C temperamental and SS impulsiveness scales. This means, that they are individuals who fear uncertainty, and at the same time are not hardworking or persistent [32]. Such profile constellation Cloninger defines as ‘disorganized’ [64,66]. Individuals with this profile have low scores on cooperativeness (C), and self-directedness (SD) scales and high scores on self-transcendence (ST) scale. The results obtained for athletes of this dispositional profile are consistent. This confirms that they have a risk-averse orientation in their behavior. They are therefore not risk takers, but also do not have developed strategies to succeed. They expect very strict instructions from their coaches, for which they try to strictly follow. However, when they are motivated by someone from the outside or they set themselves a goal (because of high scores on the transcendence scale), they do their best to achieve it [68]. They are motivated by both intense anger and euphoria [38]. As far as the aspect of impulsiveness is concerned, they are characterized by the difficulties in concentrating on a task and they have a tendency towards rash behavior when wanting to achieve an important goal [39]. This profile is also adaptive but requires a different training approach and more individualized work. Athletes of both profiles are able to be successful as long as individual training is applied.

These results only seem to be contradictory. Nowadays, the researchers of individual dispositions point out that linear models in the analysis of human personality traits (temperament) do not fully explain the actual inclinations for specific behavior, thinking or emotional reaction [69,70,71,72]. Linear models make it possible to infer a person’s personality structure and predict his or her behavior only in a very general way. In fact, temperamental and character traits are a group of dynamic intrapsychic processes through which a person learns to adapt to different life situations [72,73,74]. Meanwhile, linear explanations of human behavior do not take into consideration the various experiences (including athletes) brought from social interactions that may be moderators of behavior, thinking and decision making, both over time and in different situations [75,76].

### Limitations and Future Research Directions

Certain limitations of this study should be kept in mind for further research. For the anonymity of the participants, and to make it difficult to identify individuals on the basis of the questionnaires, questions concerning sport achievements were omitted. In future research, external criteria such as major athletic achievements, experienced injuries or the quality of relationships with other athletes should be taken into consideration. Future studies should consider comparing the profile of short track athletes with other, similar disciplines such as swimming. As only the Polish national team members (i.e., the best ones) were examined, it should be remembered that the results can be generalized only to the athletes at a high sport level. In future, similar research should be carried out among other athletes of the discipline (at lower levels), which would also increase the number of subjects. The obtained data should be compared.

## 5. Conclusions

The results of this investigation proved the hypothesis that short track athletes are characterized by specific psychological traits. Short track skaters obtain high scores on the temperamental scales i.e., persistence, harm avoidance and novelty seeking, character scales, i.e., cooperativeness and impulsivity scales, i.e., sensation seeking and positive urgency. After the cluster analysis, two homogeneous profiles of short track athletes were determined. The first profile includes athletes with high scores on the reward dependence (RD), persistence (P), self-directedness (SD), cooperativeness (C), temperamental and sensation-seeking (SS) impulsiveness scales. Low scores on the temperamental scale, harm avoidance (HA) and impulsiveness scales: positive urgency (PU), negative urgency (NU) and lack of perseverance (LP). The second profile is the reverse of the first profile. These are the athletes with high scores on the HA temperamental scale and on the PU, NU and LP impulsiveness scales. They have low scores on the RD, P, SD, C, temperamental and SS impulsiveness scales.

The research allows the determination of the psychological predispositions of short track athletes, which can be used by Schools of Sports Championships. The practical effect of the research is the possibility of setting directions for a personalized approach to the athletes in the process of training and their psychological preparation. The research results will be an element of the training process, which will be developed in the following years.

## Figures and Tables

**Table 1:** Basic descriptive statistics of short track athletes in relation to TCI-R (56) scales and S-UPPS-P scales.

Scale	Male (N = 19)						Female (N = 21)						Whole Sample (N = 40)							
	N	Mean	Min	Max	SD	N	Mean	Min	Max.	SD	t.*p*	η²	N	Mean	Min	Max	SD	S	K	K-S *p*
TCI HA raw score	19	21.5	12	27	4.91	21	26.2	17	37	5.2	2.94 0.04	0.18	40	24.1	12	37	5.5	−0.10	0.24	0.17 0.21
TCI HA sten score	19	4.2	1	6	1.4	21	5.8	3	9	1.5			40	23.9	15	34	4			
TCI NS raw score	19	23.3	19	28	2.8	21	24.5	15	34	4.8	1.03 0.31	0.02	40	24.1	16	32	4.2	0.22	0.41	0.13 0.2
TCI NS sten score	19	4.8	3	7	1.2	21	5.2	1	10	2.12			40	27.4	16	36	4.4			
TCI RD raw score	19	23.2	16	32	4.3	21	24.9	16	31	3.9	1.33 0.18	0.04	40	27.3	18	40	5.7	−0.14	−0.87	0.18 0.15
TCI RD sten score	19	4.6	2	8	1.5	21	5.2	2	8	1.4			40	25.9	15	35	5.1			
TCI P raw score	19	27.5	16	36	4.7	21	27.2	21	36	4.3	−0.21 0.83	0	40	24.6	16	36	4.9	0.01	0.01	0.08 0.2
TCI P sten score	19	4.6	1	8	1.7	21	4.6	2	8	1.4		0	40	10.9	6	16	2.2			
TCI C raw score	19	25.4	18	34	5	21	29	19	40	5.8	2.07 0.04	0.11	40	9.8	5	15	2.3	−0.00	−0.93	0.14 0.2
TCI C sten score	19	4.5	2	7	1.5	21	5.8	3	9	1.7			40	8.1	4	14	2.6			
TCI SD raw score	19	25.6	15	35	5.6	21	26.2	15	32	4. 7	0.37 0.71	0	40	7.6	4	12	2.3	−0.49	−0.52	0.11 0.2
TCI SD sten score	19	4.5	1	8	1.9	21	5	1	8	1.6			40	11.8	6	16	2.5			
TCI ST raw score	19	25.5	19	35	4.6	21	23.8	16	36	5	−1.15 0.25	0.03	40	24.1	12	37	5.5	0.65	0.2	0.12 0.2
TCI ST sten score	19	5.1	3	9	1.7	21	4.6	2	9	1.8			40	23.9	15	34	4			
UPPS NU. raw score	19	10.6	6	16	2.5	21	11	8	16	2	0.58 0.56	0	40	24	16	32	4.2	0.28	0.15	0.12 0.2
UPPS NU. sten score	19	4.9	1	9	1.7	21	4.8	2	9	1.4			40	27.4	16	36	4.4			
UPPS PU raw score	19	10.1	5	15	2.7	21	9.5	6	13	2	−0.64 0.51	0.01	40	27.3	18	40	5.7	0.28	−0.35	0.13 0.2
UPPS PU sten score	19	5.3	1	9	1.9	21	4.8	2	7	1.3			40	25.9	15	35	5.1			
UPPS LPr raw score	19	8.1	4	14	3.1	21	8.2	4	11	2.1	0.22 0.82	0	40	24.6	16	36	4.9	−0.03	−0.25	0.11 0.2
UPPS LPr sten score	19	4.8	2	8	1.9	21	5	2	7	1.4			40	10.9	6	16	2.2			
UPPS LP raw score	19	7.94	4	12	2.6	21	7.3	4	12	2	−0.83 0.41	0.01	40	9.8	5	15	2.3	0.26	−0.61	0.17 0.21
UPPS LP sten score	19	5.1	2	8	3.5	21	4.8	2	8	1.4			40	8	4	14	2.6			
UPPS SS raw score	19	11.5	8	16	2.3	21	12.2	6	16	2.6	0.97 0.33	0.02	40	7.6	4	12	2.3	−0.44	−0.41	0.17 0.21
UPPS SS sten score	19	4.5	2	8	2.5	21	5.1	1	8	1.7			40	11.8	6	16	2.5			

Note: S-skewness; K-kurtosis; K-S-Kolmogorov–Smirnov; TCI-Temperament and Character Inventory; UPPS-Urgency, Premeditation (lack of), Perseverance (lack of), Sensation-Seeking, Positive Urgency, Impulsive Behavior Scale; HA-harm avoidance; NS-novelty seeking; RD-reward dependence; P-persistence; C-cooperativeness; SD-self-directedness; ST-self-transcendence; NU-negative urgency; PU-positive urgency; LPr-lack of premeditation; LP-lack of perseverance; SS-sensation seeking.

**Table 2:** Correlation between the TCI-R (56) scales and S-UPPS-P scales.

	UPPS NU.	UPPS PU	UPPS LPr	UPPS LP	UPPS SS
TCI HA	0.24	0.014	0.08	0.17	−0.31 *
TCI NS	0.19	0.17	0.46^	0.12	0.21
TCI RD	−0.11	−0.18	0.04	−0.04	−0.05
TCI P	−0.33 **	−0.23	−0.29	−0.48^	0.47 ^
TCI C	−0.29	−0.42^	−0.03	−0.21	0.26
TCI SD	−0.37 ***	−0.36	−0.06	−0.28	0.41 **
TCI ST	0.19	0.33 ***	−0.14	−0.13	0.18

Note: TCI-Temperament and Character Inventory; UPPS-Urgency, Premeditation (lack of), perseverance (lack of), Sensation-Seeking, Positive Urgency, Impulsive Behavior Scale; HA-harm avoidance; NS-novelty seeking; RD-reward dependence; P-persistence; C-cooperativeness; SD-self-directedness; ST-self-transcendence; NU-negative urgency; PU-positive urgency; LPr-lack of premeditation; LP-lack of perseverance; SS-sensation seeking. * *p* = 0.05, ** *p* = 0.03, *** *p* = 0.02, ^ *p* = 0.001.

**Table 3:** Statistical measures of temperamental, character and impulsiveness profiles.

					Statistical Significance of Differences between Means	
Scale	Profile 1		Profile 2			
	Mean	SD	Mean	SD	F	*p*
TCI HA	−0.272	1.063	0.333	0.826	3.903	0.055
TCI NS	0.103	1.165	−0.126	0.765	0.519	0.475
TCI RD	0.363	1.006	−0.444	0.812	7.568	0.009
TCI P	0.620	0.806	−0.758	0.619	35.456	<0.001
TCI C	0.677	0.633	−0.828	0.691	51.504	<0.001
TCI SD	0.669	0.539	−0.818	0.803	48.735	<0.001
TCI ST	−0.142	0.766	0.174	1.229	0.994	0.324
UPPS NU	−0.324	1.000	0.396	0.869	5.759	0.021
UPPS PU	−0.324	0.855	0.397	1.042	5.793	0.021
UPPS LPr	−0.162	0.816	0.198	1.181	1.305	0.260
UPPS LP	−0.409	0.725	0.499	1.078	10.084	0.002
UPPS SS	0.456	0.786	−0.498	1.012	9.083	0.002

Note: TCI-Temperament and Character Inventory; UPPS-Urgency, Premeditation (lack of), Perseverance (lack of), Sensation-Seeking, Positive Urgency, Impulsive Behavior Scale; HA-harm avoidance; NS-novelty seeking; RD-reward dependence; P-persistence; C-cooperativeness; SD-self-directedness; ST-self-transcendence; NU-negative urgency; PU-positive urgency; LPr-lack of premeditation; LP-lack of perseverance; SS-sensation seeking.

## Data Availability

Not applicable.
